# History of Biological Databases, Their Importance, and Existence in Modern Scientific and Policy Context

**DOI:** 10.3390/genes16010100

**Published:** 2025-01-18

**Authors:** Mikołaj Danielewski, Marlena Szalata, Jan Krzysztof Nowak, Jarosław Walkowiak, Ryszard Słomski, Karolina Wielgus

**Affiliations:** 1 Department of Pediatric Gastroenterology and Metabolic Diseases, Poznan University of Medical Sciences, Szpitalna 27/33, 60-572 Poznan, Poland; mikolaj.danielewski@student.ump.edu.pl (M.D.); jan.nowak@ump.edu.pl (J.K.N.); jarwalk@ump.edu.pl (J.W.); 2 Department of Biochemistry and Biotechnology, Poznań University of Life Sciences, Dojazd 11, 60-632 Poznan, Poland; marlena.szalata@up.poznan.pl; 3 Institute of Medical Sciences, College of Social and Media Culture in Torun, św. Józefa 23/35, 87-100 Toruń, Poland; ryszard.slomski@aksim.edu.pl

**Keywords:** DSI, COP16, biological databases, DNA, genome

## Abstract

With the development of genome sequencing technologies, the amount of data produced has greatly increased in the last two decades. The abundance of digital sequence information (DSI) has provided research opportunities, improved our understanding of the genome, and led to the discovery of new solutions in industry and medicine. It has also posed certain challenges, i.e., how to store and handle such amounts of data. This, coupled with the need for convenience, international cooperation, and the possibility of independent validation, has led to the establishment of numerous databases. Spearheaded with the idea that data obtained with public funds should be available to the public, open access has become the predominant mode of accession. However, the increasing popularity of commercial genetic tests brings back the topic of data misuse, and patient’s privacy. At the previous United Nations Biodiversity Conference (COP15, 2022), an issue of the least-developed countries exploiting their natural resources while providing DSI and the most-developed countries benefitting from this was raised. It has been proposed that financial renumeration for the data could help protect biodiversity. With the goal of introducing the topic to those interested in utilizing biological databases, in this publication, we present the history behind the biological databases, their necessity in today’s scientific world, and the issues that concern them and their content, while providing scientific and policy context in relation to United Nations Biodiversity Conference (COP16, 21.10—1.11.24).

## 1. Introduction

The discovery of Sanger’s sequencing method in 1977 and the introduction of polymerase chain reaction (PCR) in 1983 laid the foundations for modern molecular biology. Both methods were significant improvements to the field, being far simpler and more accurate and efficient than previously used techniques. Sanger’s sequencing method, alternatively called the dideoxy method, is, as the name suggests, a laboratory technique for sequencing DNA particles. In the context of genetic analyses, ‘sequencing’ means discerning the exact sequence of nucleotides of which the specific DNA particle is composed. The purpose of PCR is the amplification of selected DNA fragments. The combination of these two strategies allowed for the generation of a huge amount of data across countless scientific projects, with the most prominent being the Human Genome Project (HGP) finished in 2003. In that endeavor, for the very first time in history, a full human reference genome was sequenced. As the samples used in the project originated from multiple individuals, the resulting genome was considered more as a representation of a typical human genome rather than a genome of a specific individual [1]. Since the human genome has around 3.2 Gbp (giga base pairs), a complete genome would require at least 3 gigabytes of storage space after file compression. Given the large scale of the human genome and taking into account all intermediate files, annotations, and genomic features, the huge storage requirements posed a substantial challenge. Another problem was data sharing. In 1991, this was principally accomplished through mailing tapes or CD-ROMs, or alternatively by downloading a larger database in multi-user centers within universities or companies and sharing them locally with many users [2]. Addressing these kinds of issues was the very reason for the creation of various databases like the National Center for Biotechnology Information (NCBI) and GenBank. However, establishing databases for storing genomic information was not without its own problems. Firstly, there was the issue of data structure—how the data are organized can impact accessibility, data integrity, how advanced querying is handled, and whether complex data with unclear relationships can be properly stored. Secondly, it was important to ensure a certain level of standardization and compatibility in communication protocols so that anyone can access the data, but also in a data format so that various databases could be interconnected, in language syntax, and in how genetic map data are represented graphically for the convenience of end user [2].

The creation of big biological databases at NCBI, the European Bioinformatics Institute (EMBL—EBI), or the DNA Data Bank of Japan (DDBJ) made it easier not only to store vast amounts of data, but also to share them internationally. As of 1991, those three databases collected sequences belonging to over 2500 organisms with the collective length of over 50 million base pairs [2]. The growth in those databases was very rapid from the start, and the rate only accelerated in the following years. In 2001, EMBL contained 6.7 million entries with a length of 8255 million base pairs. Exactly one year later, EMBL had over 12 million entries with a combined length of 12,820 million base pairs [3]. In 2004, GenBank (administrated by NCBI, see the Section 2 ‘Databases and bioinformatic tools’) contained over 37.3 million sequences, altogether 41.8 billion base pairs long. This staggering rate of growth was mainly due to many sequencing projects which strived to ascertain genomic sequences of all possible organisms; from 2003 to 2004 alone, over 50 complete microbial and 20 eukaryote genomes were deposited into GenBank [4]. As of 2024, GenBank contains sequences from 557,000 species, and altogether 3.7 billion sequences of a total length of 25 trillion base pairs [5].

During the span of the Human Genome Project, the produced data were continuously uploaded to GenBank. At first, this was mostly performed on a yearly basis so that the laboratory could obtain competitive scientific advantage from the efforts put into the sequencing process [2], but in February 1996, the international sequencing community to which the HGP centers belonged adopted a policy which stated that human sequences over 2 kbp long should be published within a 24 h period of generation. This policy has been expanded in 1998 to include all other organisms [6,7]. The HGP was an international effort, partially thanks to the spread of the Internet and the broad availability of GenBank [2,7]. The project was shared between 20 centers in 6 countries, as follows: the United States, the United Kingdom, Japan, France, Germany, and China. By 2001, they have released a draft of the nearly complete human genome. The human genome was believed to be such a precious scientific resource that from the moment of the inception of HGP, it was decided that it must be made entirely public so that it could stimulate the research benefitting human health for the greater good of the public which had founded the HGP [6].

It is not an overstatement to say that the creation of public databases and the policies accompanying the HGP were the very foundations for today’s policy of open access and open data sharing, which thus far have significantly benefitted the scientific community. Recently, however, at COP15 (15th Conference of the Parties), the United Nations Biodiversity Conference, it was noted that poor countries exploit their resources producing DSI (which are made public through the databases in accordance to the open data sharing policy), while the rich countries are benefitting from this to a disproportionate degree. As this goes against the rule from the Nagoya Protocol on Access and Benefit-sharing, this issue was widely discussed in 2024’s United Nations Biodiversity Conference (COP16) [8].

## 2. Databases and Bioinformatic Tools

Biological databases are currently indispensable tools in the majority of projects from the areas of medicine, biotechnology, molecular biology, etc. They are not only the final step in the scientific process, as a means of storing and sharing the data, but also the very first step in the scientific discovery pipeline, enabling further work. They are commonly used during the planning stage where the details of a project take shape. Biological databases store not only nucleotide sequences, but also sequences of amino acids, data regarding methylation and CpG island location, location of SNPs, list of genetic variations that have medical relevance, gene location and function, gene homologues, genetic expression data, and even the literature data.

The most important and also the most commonly known biological databases are provided by NCBI, EMBL, and DDBJ. Those entities formed the International Nucleotide Sequence Database Collaboration (INSDC) in 1986 (initially, GenBank fulfilled NCBI’s role, but this changed after the establishment of NCBI). Its purpose is to ensure that all nucleotide sequence data generated worldwide are made freely and publicly available to everyone. In order to achieve that the three databases are automatically synchronized on a daily basis, ensuring that everything that is uploaded to one of them is shared to the other two [9,10]. INSDC gathers raw sequence reads and alignments in read archives, assembled sequences with functional annotations, along with the metadata describing their origin—data regarding the biological sample (taxonomic information, tissue type), and the project for which they were generated. Each of the databases provides their users with a separate set of tools for accessing, submission, and analysis of sequence data [10].

NCBI was created in 1988 as a part of the National Institutes of Health (NIH) in the United States with the purpose of providing public biomedical databases and developing software tools for analyzing genomic and molecular data. In 1992, NCBI assumed full responsibility for GenBank, and collaborated with EMBL and DDBJ in its development. As of now, NCBI maintains over 40 integrated databases available to the public free of charge [11,12]. Below, we briefly describe the history and functionality of selected databases, a list of which can be found in Supplementary Table S1. We selected only those databases that are affiliated with NCBI or are on the list of repositories accepted by *Nature* [13]. As databases serve a variety of purposes in many different domains of science, we have divided them into broadly defined groups for ease of browsing.

In the category of chemistry-aligned databases, we have the Crystallography Open Database (COD), PubChem BioAssay, and Biological Magnetic Resonance Data Bank (BMRB). COD stores crystal structures of almost all chemical compounds, with the sole exclusion of biopolymers. It was developed by Nick Day in 2003 at the Department of Chemistry at the University of Cambridge [14]. PubChem BioAssays focuses on data regarding small molecules and RNAi. It contains information about bioactivity, toxicity, structure, and each experiment has to classify whether the reported molecule was active or inactive under given conditions. PubChem BioAssay was established in 2004 by NCBI [15]. BMBR contains data relating to metabolites and macromolecules of biological origin obtained via nuclear magnetic resonance spectroscopy. It is hosted by UConn Health (UCHC) and was developed and maintained by the University of Wisconsin in the past, starting from 1988 [16].

The Electron Microscopy Databank (EMBD) is a repository for imaging data of subcellular structures and complexes of macromolecules, obtained through electron microscopy or tomograms. It was founded in 2002 by EMBL-EBI. Since 2007, it is co-administered by the Research Collaboratory for Structural Bioinformatics (RCSB), and since 2013, also by the Protein Data Bank Japan (PDBj). In 2021, EMBD joined the Worldwide Protein Data Bank (wwPDB) organization as a member, and operates under it [17]. Neuroimaging Informatics Tools and Resources Collaboratory (NITRC) offers the three main services of resource registry, where bioinformatic tools and resources useful in neuroimaging are gathered; image repository, where neuroimaging data are stored; and computational environment. NITRC was made available in 2007, and is on the list of A NIH-Supported Scientific Data Repositories [18]. OpenNeuro is a database established in 2017 for sharing and validating MRI, PET, MEG, EEG, and iEEG data concerning the brain, that complies with Brain Imaging Data Structure (BIDS) standards [19]. The Cancer Imaging Archive (TCIA) hosts de-identified images of cancer, funded by the US National Cancer Institute, and managed by the Frederick National Laboratory for Cancer Research (FNLCR). The data are sorted by the type and location of the cancer, imaging technique, treatment and treatment outcomes, genomics, and analyses of experts [20].

The Environmental Data Initiative (EDI) is a project made in 2013 in collaboration between the University of New Mexico and the University of Wisconsin—Madison, Center for Limnology. It stores environmental data from field stations, individual laboratories, and scientific projects [21]. The Global Biodiversity Information Facility (GBIF) is an international database for storing biodiversity data on all organisms on Earth funded by the world’s governments. The collected data stem from many different sources ranging from DNA barcodes to smartphone photos [22]. KNB: The Knowledge Network for Biocomplexity is another ecology-focused database that was launched in 1998, for amassing both ecological and environmental data. It focuses heavily on metadata which provide context for interpreting complex ecological data [23]. The Terrestrial Ecosystem Research Network (TERN) Data Discovery Portal is a repository supported by the Australian Government through the National Collaborative Research Infrastructure Strategy, NCRIS. It provides open access to terrestrial ecosystem data from Australia since 2009 [24].

The EPD (Eukaryotic Promoter Database) is, as the name suggests, a database which catalogues promoter sequences for organisms of Eukaryotic origin. The transcription start sites have been determined experimentally and the annotation data include mapping data for the site, cross-references with other databases, and references from the literature. It is confined to the promoters of RNA polymerase II [25,26]. Gene is a database which stores information regarding genes, their names, unique sequences, genomic positions, expression, function, structure, and homology [12,27]. The Gene expression Omnibus, made available in 2000 and administered by NCBI, stores genomic and transcriptomic data from both array- and sequence-based approaches. As the name suggests, it focuses on quantitative data that allow for the measurement of gene expression [28]. Online Inheritance In Man (OMIM) is a database that was first published in 1966 as a physical catalog of 1487 Mendelian disorders by Victor A. McKusick under the name Mendelian Inheritance in Man. OMIM became available on the Internet with the efforts of the Welch Medical Library at the Johns Hopkins University School of Medicine, and with financial support from the Howard Hughes Medical Institute. It gathers information regarding genes, phenotypes, and the relationship between them, allowing for the search for medically significant genes [29].

GenBank was established in 1982 and is regarded as the largest and most frequently visited database. Its purpose is to gather and share all genetic sequences submitted by the researchers. This includes all organisms, viruses, and even nucleotide sequences of artificial origin. As of 2024, GenBank contains sequences from 557,000 species, altogether 3.7 billion sequences of total length of 25 trillion base pairs. Its growth was rapid from its very beginning and this pattern still continues, with its size doubling every 2 years [5]. GenBank is administered by the NCBI and is one of the repositories from which the Nucleotide database takes its data. Genome is a database which organizes and groups all sequencing projects based upon the organism analyzed [12]. Nucleotide is a database storing nucleotide sequences from many different sources, but predominantly from GenBank, and RefSeq [12]. RefSeq was established by NCBI in 1999. It provides and gathers reference sequences for genomes, transcripts, and proteins for viruses, microorganisms, organelles, and eukaryotic organisms. Unlike GenBank, which gathers all sequences that were submitted to it, RefSeq accepts only properly curated data that have been validated [30]. Sequence Read Archive (SRA) is a database which supports the storage, retrieval, and analysis of high throughput genome sequencing data. It is one of the largest biological databases, containing 11.5 Petabytes of data available to public [12].

In the broad category of health, we list the following: ClinicalTrials.gov, ImmPort, and PhysioNet. ClinicalTrials.gov is a database supported by NCBI, created in 2000, which stores information regarding clinical research studies and their results [31]. ImmPort is an immunology database and analysis portal, which focuses on gathering data pertaining to immunology, be it allergies, autoimmune diseases, or transplantation. It collects both results and protocols form clinical trials, as well as potential net methods for measuring on a cellular or molecular level [32]. PhysioNet, otherwise known as the Research Resource for Complex Physiologic Signals, was created in 1999 by the National Institutes of Health to provide access to physiological and clinical data. It stores single physiological signals and in time series, for various organs and tissues, in both healthy individuals and in patients [33].

Bookshelf was made available in 1999. It provides free access to the full text of books, reports, literature databases, and documentation regarding life sciences and medicine [12,34]. The National Library of Medicine Catalog (NLM Catalog) was introduced in 2004 to provide bibliographic data for journals, books, audiovisuals, software, etc. [12,35]. PubMed was made public in 1996 with the intention of supporting the search and retrieval of life sciences and the medically relevant literature. It contains abstracts and citations from medical and scientific publications, but it does not provide their full text. It does, however, provide the links to the journals in which the articles were published, and to full texts if they are available in Open Access [12,36]. PubMed Central (PMC) was made available to the public in 2000. Its goal is to provide free access to full text versions of medical and scientific journal literature. It also contains preprints and manuscripts submitted through NIH Manuscript Submission System. For the year 2024, 10,307,502 articles were available [37].

BioProject, made public in 2011 by NCBI, stores detailed information regarding research projects that are the sources for submissions in other NCBI databases. This ties together biological samples and datasets, providing better clarity. It also serves as a platform to inform about data availability [38]. Biosamples was created in 2011 to provide information regarding the samples from which the data in other NCBI databases were derived [12,39]. Datasets is a recent database, created by NCBI to more easily provide access to metadata and sequence data available in NCBI databases. It allows for downloading large datasets, together with metadata, as opposed to downloading multiple separate positions and forming a dataset yourself. As of June 2024, it has replaced the legacy Genome and Assembly web resources. The contents of both Gene and Genome have been integrated into Datasets, and are now accessible through that platform [40]. MetaboLights gathers metabolomic data including the structure of metabolites, their locations, and concentrations. It accepts data from different species and generated using various approaches [41].

Mouse Genome Informatics (MGI) is an international database created in 1989 for sharing data regarding laboratory mice as a model organism. It stores genetic, genomic, and biological data for the study of human health and disease [42]. The Rat Genome Database (RGD) was established in 1999 as a repository for data derived from rat research, which includes genetics, genomics, phenotypes, and diseases. Currently, it stores data on the following ten different species: rat, mouse, human, chinchilla, bonobo, 13-lined ground squirrel, dog, pig, green monkey/vervet, and naked mole-rat [43]. FlyBase is a database intended for storing and sharing the genes and genomes of *Drosophila*. The scope includes genes, alleles, phenotypes, aberrations, clones, and stock lists [44,45].

EBRAINS is a repository for all data, tools, and computing facilities for brain research, funded in 2019 as a result of the Human Brain Project [46]. NeuroMorpho.org gathers centrally curated images of digitally reconstructed neurons and glia. It is currently the largest collection of publicly available 3D reconstructions of neurons [47].

In this paragraph, we include all the databases from the “Other” category from Supplementary Table S1, as they could not be grouped thematically with the other databases. The BioModels Database, created in 2005, gathers and shares mathematical models of biological and biomedical systems. The models, based on the literature, are designed for studies of physiology and pharmaceutics [48]. FlowRepository, made available in 2012, a is database intended for sharing the results of cell flow cytometry-based experiments [49]. Taxonomy is a database that stores taxonomic names present in other NCBI databases; its purpose is to organize the data and allow for easier browsing. If a sequence belonging to as yet unknown organism is added to GenBank, the new name is properly classified and added to the Taxonomy database [12,50]. MeSH (Medical Subject Headings) is an online thesaurus for indexing, cataloguing, and searching biomedical information [12,51]. UK Data Service is a repository created in 1967, for data from the research on economy, sociology, and demography from the United Kingdom [52].

The Bacterial and Viral Bioinformatics Resource Center (BV-BRC) is a database for all data concerning viral and bacterial diseases. It was merged in 2019 from the three different databases, namely, the Influenza Research Database (IRD), the Virus Pathogen Database and Analysis Resource (ViPR), and PAThosystems Resource Integration Center (PATRIC). It includes genomes and their annotations, relevant metadata, and non-genomic data like protein structures and immune epitopes [53]. The Eukaryotic Pathogen, Vector and Host Informatics Resource (VEuPathDB) database focuses on gathering and sharing genomic and other large datasets for the organism within its thematic scope, which include pathogens of infectious diseases and their mammalian hosts, as well as invertebrate vectors [54].

PeptideAtlas, created in 2004, is a compendium of peptides from different organisms (mainly human, mouse, and yeast, but also a few others), identified through tandem mass spectrometry [55]. The Protein Circular Dichroism Data Bank (PCDDB) is a database for storing circular dichroism (CD) and synchrotron radiation CD (SRCD) spectral data from protein-related research [56]. The UniProt database was formed in 2002 by groups from the Swiss Institute of Bioinformatics, the European Bioinformatics Institute, and Information Resource at Georgetown University. Currently, UniProt is one of the main sources for sequence and annotation data for proteins. It provides tools for searching, retrieval, mapping identifiers, and aligning multiple sequences [57]. The Worldwide Protein Data Bank (wwPDB) is an organization that administers the Protein Data Bank, which stores information regarding 3D structures of large biomolecules like proteins, DNA, and RNA [58].

ClinVar is a database that was made available to the public in 2014. It catalogues all genetic variations from dbSNP and dbVar that have been noted to have clinical significance [59]. dbSNP was established in 1998 to catalog information regarding variance in the form of short nucleotide polymorphisms (SNPs). It collects the data on where in the genome the variations are present, how frequently they occur, the effects of the alleles, and the organism in which they are present [12,60]. dbVar was created in 2004 to gather the data about copy number variation, insertions, deletions, and translocations longer than 50 bp [12,61].

## 3. Databases, Open Data Sharing and Ethical Problems

As mentioned previously, the human genome generated from HGP was from the very beginning planned to be made publicly available, because of its significant importance for human health and future research [6,7]. This, coupled with the creation of many biological databases, has likely laid foundations for the idea of open access and open data sharing. Open data sharing is very beneficial for the scientific community and the prospects of future research, as it ensures that each dataset will be thoroughly scrutinized by many different scientists, each with different ideas on what to use it for. As generating datasets is rather challenging and costly in both time and money, it is important that they are used to the fullest. This rings especially true in the case of projects that are covered from public funds. Open data sharing also provides opportunity for scientists to reproduce and verify each other’s results, which is beneficial for the quality of the research. It also introduces element of international cooperation even if the original project did not plan on it, as the generated dataset can always be utilized by researchers from another country. Lastly, as the majority of projects are funded by the public, it is only natural that the public should have free access to the data they helped to generate [62].

On the other hand, genomic sequences are very sensitive data, and improper handling may result in ethical problems. When the Human Genome Project was underway, the issue of the ethical, legal, and social implications was raised. While the potential positive effects of researching the human genome were undeniable, it was also agreed that it may have many important implications for the individuals and the society. Thus, five goals were established by Francis S. Collins et al. in 1998 [6], which related to the examination of issues arising from the accomplishment of HGP and the study of human genetics, the introduction of genetics into healthcare, understanding of how environment and genetics interact (which today is very topical), the interface between genomics and philosophy, as well as the risks to equality brought about by genetic technologies.

One can easily see that those concerns are still valid more than 20 years later. However, the amount of available data is much greater, and so are the possibilities of generating it, as the prices of genome sequencing have greatly lowered. While proper anonymization of data is always rigorously exacted, with the advent of commercially available genome sequencing the once anonymous data may be linked, e.g., to relatives. Because family members naturally have large portions of genome in common, one family member having their genome sequenced inadvertently shares parts of genomes of the rest of the family, which may allow for unwanted identification.

Furthermore, researchers are not allowed to perform certain analyses on the genomes of patients without their consent (or share such results), as the patients themselves may not wish to know the result. Reporting of variants of unknown significance is a similar problem, which can be regulated by adequate guidelines. There are a number of issues which present an unaddressed grey area, potentially leading to complications in the future. This leads back to the issue of proper data storage, handling, and the protection of potentially sensitive information, policies in relation to which may need to change [63].

## 4. DSI and COP16

DSI is a term with no broadly accepted definition but it usually refers to nucleic acid sequences (genes, genomes, mRNA, etc.), amino acid sequences, information regarding the structure of nucleic acid sequences (genetic mapping, secondary, tertiary, etc., structure of nucleic acids and proteins), data regarding gene expression, data about macro-particles and cellular metabolites, ecological relationships, abiotic components of environment, functions of living organisms (i.e., data about animal behavior), structure of living organisms (population data, morphology, phenotype, etc.), applications of living organisms, biological components, and biological data [combined study, DSI scientific network]. As DSI is most commonly used in regard to biodiversity, this term does not include human sequences.

During COP15, it was noted that there exists an issue of not equally sharing the benefits from DSI. It was said that developing countries endanger their biodiversity by conducting research and providing DSI, while the developed countries profit from the utilization of DSI to an disproportionate degree [64]. Because of this, among many others, the following decisions were made:

(a) The term ‘DSI’ will remain in use during the talks despite its lack of clear definition;
(b) In the context of benefit sharing, the DSI will be addressed from the perspective of the Nagoya protocol—as genetic resources;
(c) It was agreed that a solution for equitable benefit-sharing needs to be developed, and the benefits from use of DSI should be used to support conservation and sustainable use of biological diversity;
(d) The form of equitable benefit-sharing will take the form of a global fund.

The Nagoya Protocol on Access and Benefit-sharing, which was agreed upon at the 10th COP in Nagoya, Japan, states that benefits from the utilization of genetic resources should be shared fairly and equitably [8].

In 2024, from 21 October to 1 November, COP16 took place, and a decision regarding DSI and benefit-sharing was made. The creation of a global fund called the Cali Fund for the Fair and Equitable Sharing of Benefits from the Use of Digital Sequence Information on Genetic Resources was announced. Users of DSI which exceed two of the three following thresholds, when averaged over three years, will be obliged to contribute 1% of their profits or 0.1% of revenue to the Cali Fund:

(a) total assets worth more than USD 20 million;
(b) Sales greater than USD 50 million;
(c) Profit greater than USD 5 million.

The contribution rates and the thresholds are not final, and will be established at COP17, and reviewed periodically afterwards. Additionally, it was emphasized that the non-monetary benefits from DSI should be shared fairly and equitably, complimentarily to the monetary benefit-sharing. What is particularly important is that public research institutions, academic institutions, and entities operating public databases are not required to make contributions to the global fund. The Cali Fund will be used for the purpose of conservation and sustainable use of biodiversity in developing countries, and especially the least-developed countries. This funding will be also made available to the indigenous people in developed countries for those very same purposes. The criteria on which the funding will be allocated will be determined at COP17. Furthermore, at least half of the funding of the Cali Fund should be used to support the needs of indigenous people and local communities [65]. While COP16 ended on an optimistic note with regards to the DSI issue, some pointed out that the United States of America did not ratify the Convention on Biological Diversity and thus is not bound by the decisions at COP16. As USA is one of the biggest users of DSI, its participation could potentially increase the Cali Fund by a large margin [66].

## 5. Conclusions

Biological databases are a crucial element in the fields of life sciences and biomedical research. While initially they were used mainly to deposit data, currently their usage is an important step in many analyses. A sequenced genome is aligned against a reference taken from a database. When analyzing a gene and its function, we may want to check where it is expressed, where in the genome it is located and what other genes are surrounding it, whether it is clinically relevant, and how many variants of this gene exist in the human population. Every single one of those questions can be answered with a relatively simple query in an appropriate database. As the field of biology will only keep becoming more data-rich, the role of bioinformatics will only keep becoming more important within it. Even in the early 1990s, it was said that the most limiting factor in fully utilizing databases will be the ignorance or the reluctance of the user, and that there should be a strong educational effort to teach computer science and genome informatics in courses related to biomedicine. This rings especially true in today’s age, when AI tools are more and more broadly used. Along with biology, informatics should be taught in equal measure, as interdisciplinary expertise is always needed, as clearly demonstrated by the emergence of the convergent field of bioinformatics [2,6,67].

The falling prices of genome sequencing and the increasing availability of commercial sequencing for the public can be a potential source of future problems of ethical nature and safety of personal information. Some AI models have already been utilized to screen job applicants, where they have been shown to be highly discriminatory, and the question is whether AI could inadvertently learn to discriminate based on the genotype [68]. Polygenic risk scores and the knowledge of individual variants strongly related to health outcomes opens questions regarding the potential use of such data in the context of insurance for individuals, families, and even populations. The imperative of considering genomic sequences of individuals as personal information has been recognized in the European Union and beyond, which has impacts on policies regarding data storage and handling.

Lastly, the creation of the Cali Fund announced at COP16 will help share benefits from DSI globally but also shifts the paradigm of DSI use towards for-profit applications. The free, unrestricted access to biological data has thus far stimulated research. Academic scientists continue to freely access crucial data while a precedent for regulating its use has been set.

## Data Availability

All data is contained within this article.

## Supplementary Materials

The following supporting information can be downloaded at: https://www.mdpi.com/article/10.3390/genes16010100/s1, Table S1: List of selected biological databases, organised alphabetically by category and name.
