# Supplementary material for: History of Biological Databases, Their Importance, and Existence in Modern Scientific and Policy Context
Source: Genes (Basel). 2025 Jan 18;16(1):100. doi: 10.3390/genes16010100 (PMC11765253; doi:10.3390/genes16010100)
Supplement: Supplementary file 1 [file genes-16-00100-s001.zip › genes-3432121-supplementary.pdf]

Supplementary Table S1. List of selected biological databases, organised alphabetically by category and name.

Name	Year of Creation	Category	Link
Crystallography Open Database (COD)	2003	Chemistry	http://www.crystallography.net/cod/
PubChem BioAssay	2004	Chemistry	https://pubchem.ncbi.nlm.nih.gov/docs/bioassays
Biological Magnetic Resonance Data Bank (BMRB)	1988	Chemistry	https://bmrb.io/
Electron Microscopy Data Bank (EMDB)	2002	Data Imaging	https://www.ebi.ac.uk/emdb/
Neuroimaging Informatics Tools and Resources Collaboratory (NITRC)	2007	Data Imaging	https://www.nitrc.org/
OpenNeuro (formerly OpenfMRI)	2017	Data Imaging	https://openneuro.org/
The Cancer Imaging Archive (TCIA)	2011	Data Imaging	https://www.cancerimagingarchive.net/
Environmental Data Initiative	2013	Ecology	https://edirepository.org/
Global Biodiversity Information Facility (GBIF)	2001	Ecology	https://www.gbif.org/
KNB: The Knowledge Network for Biocomplexity	1995	Ecology	https://knb.ecoinformatics.org/
TERN (Terrestial Ecosystem Research Network) Data Discovery Portal	2009	Ecology	https://portal.tern.org.au/
EPD (Eukaryotic Promoter Database)	1986	Genes	https://epd.expasy.org/epd/
Gene	1999	Genes	https://www.ncbi.nlm.nih.gov/gene
Gene Expression Omnibus	2000	Genes	https://www.ncbi.nlm.nih.gov/geo/
OMIM (Online Inheritance In Man)	1966	Genes	https://www.omim.org/
GenBank	1982	Genomes	https://www.ncbi.nlm.nih.gov/genbank/
Genome	-	Genomes	https://www.ncbi.nlm.nih.gov/datasets/genome/
Nucleotide	2006	Genomes	https://www.ncbi.nlm.nih.gov/nuccore
RefSeq	1999	Genomes	https://www.ncbi.nlm.nih.gov/refseq/
SRA (Sequence Read Archive)	2007	Genomes	https://www.ncbi.nlm.nih.gov/sra
ClinicalTrials.gov	2000	Health	https://clinicaltrials.gov/about-site/about-ctg
ImmPort	2004	Health	https://immport.org/shared/home
PhysioNet	1999	Health	https://physionet.org/

Booshelf	1999	Literature	https://www.ncbi.nlm.nih.gov/books
NLM (National Library of Medicine) Catalog	2004	Literature	https://www.ncbi.nlm.nih.gov/nlmcatalog/
PubMed	1996	Literature	https://pubmed.ncbi.nlm.nih.gov/
PubMed Central (PMC)	2000	Literature	https://pmc.ncbi.nlm.nih.gov/
Bioproject	2011	Metadata	https://www.ncbi.nlm.nih.gov/bioproject/
Biosamples	2011	Metadata	https://www.ncbi.nlm.nih.gov/biosample
Datasets	2023	Metadata	https://www.ncbi.nlm.nih.gov/datasets/
MetaboLights	2012	Metadata	https://www.ebi.ac.uk/metabolights/
Mouse Genome Informatics (MGI)	1989	Model Organims	https://www.informatics.jax.org/
Rat Genome Database (RGD)	1999	Model Organims	https://rgd.mcw.edu/
FlyBase	1992	Model Organisms	https://flybase.org/
EBRAINS	2017	Neuroscience	https://www.ebrains.eu/
NeuroMorpho.org	2006	Neuroscience	https://neuromorpho.org/
BioModels Database	2005	Other	https://www.ebi.ac.uk/biomodels/
FlowRepository	2012	Other	http://flowrepository.org/
MeSH (Medical Subject Headings)	1960	Other	https://www.nlm.nih.gov/mesh/meshhome.html
Taxonomy	1991	Other	https://www.ncbi.nlm.nih.gov/Taxonomy/Browser/wwwtax.cgi
UK Data Service	1967	Other	https://ukdataservice.ac.uk/
Bacterial and Viral Bioinformatics Resource Center (BV-BRC)	2019	Pathogens	https://www.bv-brc.org/
The Eukaryotic Pathogen, Vector and Host Informatics Resource (VEuPathDB)	2004	Pathogens	https://veupathdb.org/
PeptideAtlas	2004	Proteins	https://peptideatlas.org/
Protein Circular Dichroism Data Bank (PCDDB)	2009	Proteins	http://pcddb.cryst.bbk.ac.uk/
UniProt	2002	Proteins	https://www.uniprot.org/

Worldwide Protein Data Bank (wwPDB)	2003	Proteins	https://www.rcsb.org/
ClinVAr	2014	Variants	https://www.ncbi.nlm.nih.gov/clinvar/
dbSNP	1998	Variants	https://www.ncbi.nlm.nih.gov/snp/
dbVar	2004	Variants	https://www.ncbi.nlm.nih.gov/dbvar/
